# Integrating the Memory Support Intervention into the Transdiagnostic Intervention for Sleep and Circadian Dysfunction (TranS-C): can improving memory for treatment in midlife and older adults improve patient outcomes? Study protocol for a randomized controlled trial

**DOI:** 10.1186/s13063-024-08468-0

**Published:** 2024-10-03

**Authors:** Anne E. Milner, Rafael Esteva Hache, Sophia Oliver, Laurel D. Sarfan, Julia M. Spencer, Ashby Cogan, Yimei Jiang, Emma R. Agnew, Garret G. Zieve, Jennifer L. Martin, Michelle R. Zeidler, Lu Dong, Joseph K. Carpenter, Joshua Varghese, Kiely Bol, Zia Bajwa, Caitlan A. Tighe, Allison G. Harvey

**Affiliations:** 1 Department of Psychology, University of California, Berkeley, CA USA; 2 Oakland Cognitive Behavior Therapy Center, Oakland, CA USA; 3 Geriatric Research, Education, and Clinical Center, VA Greater Los Angeles Healthcare System, Los Angeles, USA; 4 Department of Medicine, David Geffen School of Medicine at the University of California, Los Angeles, USA; 5 UCLA Health Pulmonology, Los Angeles, CA USA; 6 Behavioral and Policy Sciences, RAND Corporation, Santa Monica, CA USA; 7 National Center for PTSD, Women’s Health Sciences Division, VA Boston Healthcare System, Boston, MA USA; 8 Department of Psychology, Providence College, Providence, RI USA

**Keywords:** Memory support, Transdiagnostic, Sleep, Circadian

## Abstract

**Background:**

Poor memory for treatment is associated with poorer treatment adherence and poorer patient outcomes. The memory support intervention (MSI) was developed to improve patient memory for treatment with the goal of improving patient outcomes. The aim of this study protocol is to conduct a confirmatory efficacy trial to test whether a new, streamlined, and potent version of the MSI improves outcomes for midlife and older adults. This streamlined MSI is comprised of constructive memory supports that will be applied to a broader range of treatment content. The platform for this study is the Transdiagnostic Intervention for Sleep and Circadian Dysfunction (TranS-C). We will focus on midlife and older adults who are low income and experiencing mobility impairments.

**Methods:**

Participants (*N* = 178) will be randomly allocated to TranS-C + MSI or TranS-C alone. Both intervention arms include eight 50-min weekly sessions. Assessments will be conducted at pre-treatment, post-treatment, 6-, and 12-month follow-up (6FU and 12FU). Aim 1 will compare the effects of TranS-C + MSI versus TranS-C alone on sleep and circadian functioning, daytime functioning, well-being, and patient memory. Aim 2 will test whether patient memory for treatment mediates the relationship between treatment condition and patient outcomes. Aim 3 will evaluate if previously reported poor treatment response subgroups will moderate the relationship between treatment condition and (a) patient memory for treatment and (b) treatment outcome. Exploratory analyses will compare treatment condition on (a) patient adherence, patient-rated treatment credibility, and patient utilization of treatment contents, and (b) provider-rated acceptability, appropriateness, and feasibility.

**Discussion:**

This study has the potential to provide evidence for (a) the efficacy of a new simplified version of the MSI for maintaining health, well-being, and functioning, (b) the wider application of the MSI for midlife and older adults and to the treatment of sleep and circadian problems, and (c) the efficacy of the MSI for sub-groups who are likely to benefit from the intervention.

**Trial registration:**

ClinicalTrials.gov NCT05986604. Registered on 2 August 2023.

## Introduction

### Background and rationale {6a}

Patient memory for the content of treatment is poor. Only one third of recommendations made during physician visits are accurately recalled (e.g., [1–3]), and patients may recall *in*accurate information. Indeed, one study found that 25% of patients recalled treatment recommendations that had *not* been made [4]. Patients receiving cognitive and behavioral treatments have similarly poor memory for their treatment [3, 5]. Collectively, these findings are important because poor memory for treatment has been associated with poorer adherence [6–8] and poorer outcomes [3, 7, 9].

There are several explanations for why patient memory may be poor. First, even when memory is functioning optimally, memory can fail during the encoding and retrieval process [10]. Second, cognitive and behavioral treatment sessions are long, cover complex material, and can elicit negative emotions that bias attention and in turn bias encoding of information [11]. Third, patients may also struggle to transfer knowledge learned in therapy to real-world situations, known as the transfer of learning problem; in other words, they may struggle to recall applicable treatment skills and concepts in their everyday lives [12].

Promisingly, however, the impact of memory failures can be minimized at both the encoding and retrieval stages through the use of memory support strategies, which have been shown to be effective at improving memory in individuals with depression and Alzheimer’s disease [13–15]. Building on this prior research, the Memory Support Intervention (MSI) was developed to improve patient memory for treatment contents with the aim of improving patient outcomes [16]. In line with the Experimental Therapeutics Approach [17, 18], the MSI was designed to engage a target mechanism of change, *patient memory for treatment*, which is proposed to mediate patient outcomes [16]. The intervention was designed to improve memory for treatment contents rather than to improve general memory functioning. The original MSI consists of eight different memory supports that were derived from education and cognitive psychology research [19–22]. Four of the memory support strategies are *constructive*, defined as supports that prompt patients to generate new ideas, inferences, or connections in relation to treatment content. The other four memory support strategies are *non-constructive* and highlight aspects of treatment without asking patients to generate anything new related to it [16, 23]**.**

In a small pilot study (*n* = 48), adults with major depressive disorder (MDD) were randomized to receive either cognitive therapy plus the Memory Support Intervention (CT + MSI) or cognitive therapy as usual (CT-as-usual) [24]. Patient memory for treatment contents was enhanced in the CT + MSI condition relative to CT-as-usual at post-treatment with a small to medium effect size. Results from a confirmatory efficacy trial (*N* = 178) where patients diagnosed with MDD were also randomized to CT + MSI or CT-as-usual found improvements in the CT + MSI condition relative to CT-as-usual on only two secondary outcome measures: (1) lower depression severity at 6-month follow-up and (2) a greater reduction in unhealthy days from pre-treatment to 6-month follow-up [25]. A secondary analysis of this trial assessed differences at 12-month follow-up and did not find any significant differences in outcomes between the two treatment conditions [26].

There are several lines of evidence from other secondary analyses that indicate additional investigation is needed. First, the dose of memory support delivered in the trial may not have been sufficient to elicit significant improvements in patient memory or treatment outcomes [9]. Second, therapist use of memory support was indirectly associated with improved patient outcomes. Specifically, this effect was mediated by greater patient adherence to treatment recommendations as well as increased utilization and competency of CT skills, up to 1 year after treatment [27]. Third, therapists used most of their memory support for cognitively focused treatment contents including the cognitive model and cognitive restructuring which resulted in patients having improved recall of this information [26]. The latter point is of concern because it has been demonstrated that *procedural* treatment contents (e.g., practical intervention recommendations) are more closely related to patient outcomes than *conceptual* treatment contents (e.g., the underlying theoretical model used in treatment) [28]. Therefore, it is possible that greater therapist use of MS for procedural treatment content (e.g., behavioral activation) may be more effective than conceptual treatment content (e.g., the cognitive model). Training therapists to more adequately spread their use of memory support to a broader range of treatment content could strengthen the MSI. Fourth, constructive memory support strategies have been demonstrated to be more effective at improving patient recall relative to non-constructive strategies [23]. However, in prior research, there was not a sufficient focus on constructive memory support strategies, with therapists delivering non-constructive strategies twice as frequently as constructive [29]. Similarly, in the pilot study testing the MSI, therapists used non-constructive strategies four times more frequently than constructive strategies [30]. Given that constructive strategies appear to be more effective at improving memory for treatment, focusing on constructive memory support strategies may further strengthen the MSI. Taken together, these findings indicate that the MSI may engage important treatment mechanisms (e.g., adherence, skill competence, and utilization) that indirectly improve outcomes but could benefit from further investigation to engage the target of patient memory more effectively, which in turn, has potential to enhance the direct effect on patient outcomes [17].

In the paragraphs that follow, we will discuss how the present study will expand the evidence base on the MSI and test changes to increase its potency. The MSI was developed to be applied to a broad range of treatments (“pantreatment”) and to different mental disorders (“transdiagnostic”). As reviewed above, the MSI has so far only been tested for one mental disorder (major depressive disorder) and one type of treatment (CT) [25]. CT has been demonstrated to be an efficacious treatment that already has some memory support strategies embedded [31]. Therefore, further research is needed to evaluate the utility of memory support beyond CT alone, at a higher dose, and for a wider range of treatment content.

In the current study, the MSI has been streamlined to include only the most potent memory supports. The MSI was originally comprised of 8 different memory supports but has been simplified to include only the 4 *constructive* memory supports that prompt learners to generate new ideas, inferences, or connections that go beyond what is explicitly presented in treatment-as-usual. Findings have suggested that these strategies result in better learning, relative to passively absorbing information [23].

The current study also builds on prior research by investigating a broader application of the MSI. Specifically, we will recruit midlife and older adults who are experiencing problems related to sleep or circadian functioning. We focus on midlife and older adults for several reasons. As life expectancy increases, so does the aging population [32, 33]. Healthy aging is associated with declines in memory functioning [34–38]. This decline in memory functioning occurs during a period of life when there is often an increased need to utilize health services [39]. Considering evidence for the relationship between poor memory for treatment and poor treatment outcomes, coupled with reduced memory functioning and increased health care utilization in older individuals, improving patient memory for treatment, via the MSI, has been hypothesized to be highly impactful for midlife and older individuals [16].

The “platform” through which we will continue studying the MSI is a psychological treatment for sleep and circadian problems. This focus was selected because sleep and circadian disturbance is common among midlife and older adults [40–42]. For example, healthy aging is associated with decreases in total sleep duration, decreased sleep efficiency, increased sleep latency, and more frequent nighttime awakenings [43, 44]. Untreated sleep and circadian problems also have critical negative consequences including an increased risk for new onset of psychiatric and medical illness [45], increased accidents [46, 47], increased fall risk [48–50], increased chronic pain [51], and reduced physical activity [52].

Cognitive behavior therapy for insomnia (CBT-I) has been demonstrated to be an effective treatment for insomnia among midlife and older adults [53, 54] while also improving the symptoms of comorbid physical and mental disorders [55–58]. However, midlife and older adults experience a broader range of sleep and circadian dysfunctions (e.g., advanced circadian phase, medical problems, sleep apnea). To address this broader range, the current research will focus on the Transdiagnostic Sleep and Circadian Intervention (TranS-C) [59] as it incorporates the components of CBT-I, but also includes a range of content related to other sleep and circadian problems experienced by midlife and older adults. TranS-C is a modular, skills-based approach that is transdiagnostic in that it has been designed to address a range of sleep and circadian problems for a range of mental and physical disorders. Interestingly, in prior research, older people had a poorer response to TranS-C relative to younger people [60]. In another study limited to midlife and older adults, TranS-C was associated with improvements in many outcomes (depression, sleep disturbance, sleep health, and select sleep/wake outcomes), but not all improvements were sustained at follow-up [61]. The present study tests whether adding the MSI to TranS-C may further improve outcomes for midlife and older people.

Previous research has also demonstrated other sub-groups that have a poor response to treatment including individuals with fewer years of education [24, 62, 63], poorer baseline cognitive functioning [64, 65], and greater baseline symptom severity [66]. We will also examine if these poor response sub-groups will derive a particular benefit from adding the MSI to TranS-C.

From this population of midlife and older adults, we will also focus on recruiting individuals who are low-income and experience mobility impairments. We selected this focus as these are two problems that co-occur with aging [41, 67] yet are minimally studied [68–70]. Furthermore, there is an unmet need for health care for individuals who are both low-income [71–73] and have mobility impairments [71–73]. There is increasing scientific evidence supporting the use of telehealth in sleep and circadian medicine [74–76]. Therefore, the current study will deliver the MSI via telehealth to increase accessibility for this underserved population.

In summary, the overarching goal of the current study is to assess whether memory support strategies implemented into TranS-C can improve patient memory for treatment thereby improving patient outcomes among midlife and older adults. Over a 4-year period, midlife and older adults (*n* = 178, including 20% for attrition) will be randomly allocated to TranS-C + MSI or TranS-C alone and will be assessed at pre-treatment, post-treatment, 6-month follow-up (6FU), and 12-month follow-up (12FU). This is a randomized control trial that incorporates aspects of both Stages 2 and 3 of the NIH Stage Model [77]. Specifically, therapists affiliated with the research team will deliver the intervention to maintain the high level of control necessary to establish fidelity and internal validity (Stage 2). Participants will be recruited from community settings as a first step toward testing the delivery of the MSI in routine practice (Stage 3), bridging the gap between research and practice [78, 79].

### Objectives {7}

The first aim is to assess the efficacy of incorporating the MSI into TranS-C, compared to TranS-C alone. We hypothesize that compared to TranS-C alone, TranS-C + MSI will be associated with improvements in sleep and circadian functioning, daytime functioning, well-being, and patient memory at post-treatment, 6FU, and 12FU. The second aim is to evaluate if patient memory for treatment contents mediates the relationship between treatment condition and sleep and circadian functioning. We hypothesize that TranS-C + MSI will be associated with enhanced memory for treatment relative to TranS-C alone and enhanced memory for treatment will be associated with improvements in sleep and circadian functioning at post-treatment, 6FU, and 12FU. The third aim is to assess whether previously reported poor treatment response sub-groups will moderate the relationship between treatment condition and (a) patient memory for treatment and (b) treatment outcome, such that individuals in these sub-groups may derive a greater benefit from receiving memory support. It is hypothesized that treatment effects for those in the TranS-C + MSI condition will be larger at post-treatment for individuals who are older, have fewer years of education, poorer baseline cognitive functioning, and more severe baseline sleep disruption and sleep-related impairment. Exploratory analyses will compare treatment condition on (a) patient adherence, patient-rated treatment credibility, and patient utilization of treatment contents and (b) therapist-rated acceptability, appropriateness, and feasibility.

### Trial design {8}

This study is a parallel group, randomized, controlled, superiority trial. Participants will be randomized to TranS-C + MSI or TranS-C alone with a 1:1 allocation ratio.

## Method: participants, interventions, and outcomes

**Fig. 1:**
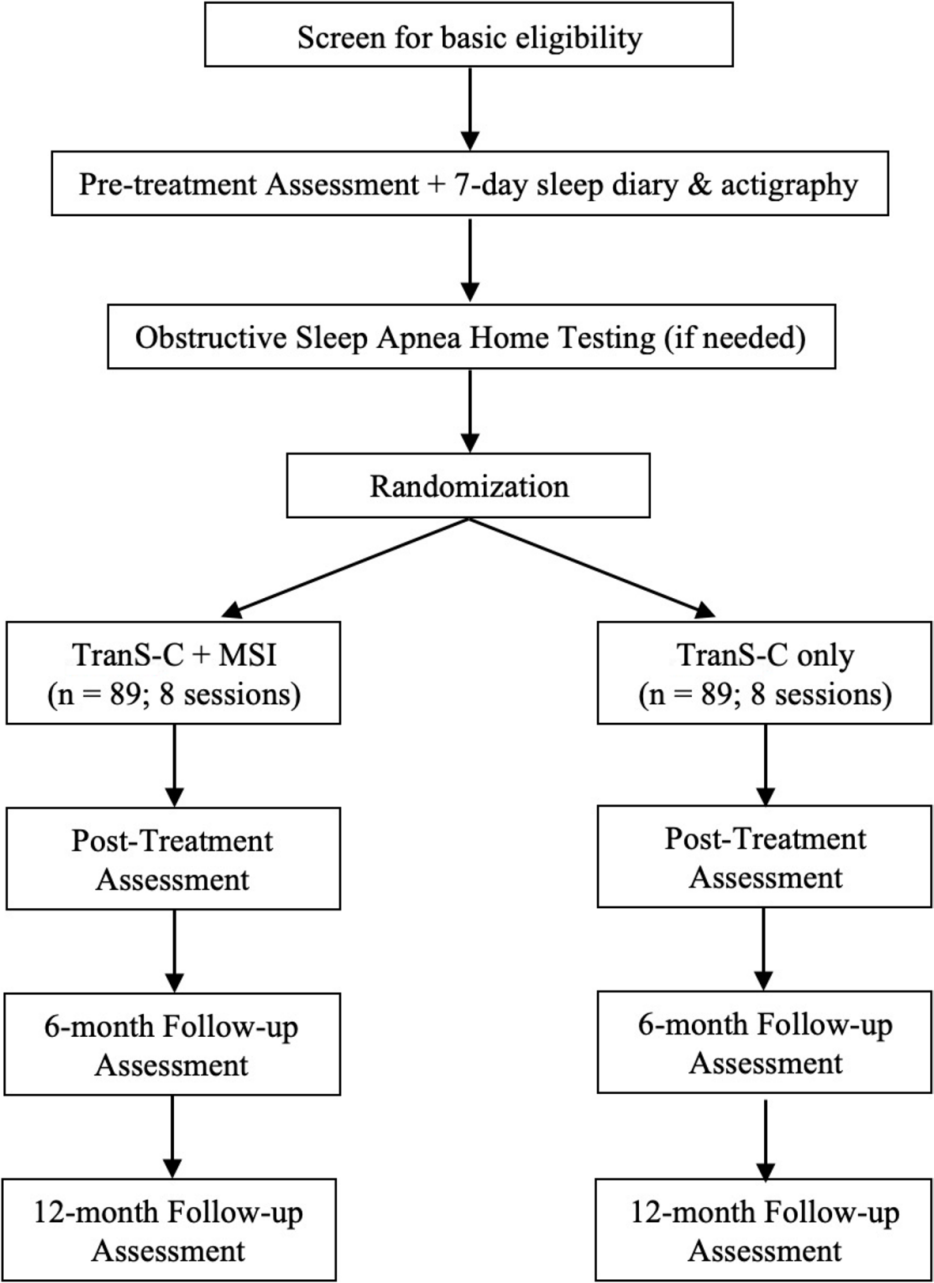
Standard Protocol items: anticipated patient flow for randomized clinical trial

This study was preregistered on clinicaltrials.gov (identifier: NCT05986604). The study received approval from the Committee for the Protection of Human Subjects (CPHS) at the University of California, Berkeley (UCB). If there are too many findings to reasonably interpret in one paper, we may separate some of the findings into multiple papers. This research is funded by the National Institute of Aging (R01AG082651). See Fig. 1 for a flow diagram depicting the study design.

### Study setting {9}

A total of 178 adults who meet the eligibility criteria will be recruited within the USA. As sleep problems tend to be more prevalent in females relative to males [80, 81] and females tend to live longer than males [82], we plan to overrecruit females (65% females/35% males). Participants will be reimbursed $100 for each of the three post-treatment assessments, for a total of $300.

### Eligibility criteria {10}

#### Inclusion criteria

The inclusion criteria are:

- Aged 50 years and older
- English language fluency
- Experiencing mobility impairment as determined by endorsing “mild”, “moderate”, “severe”, or “extreme or cannot do” on at least one item on the WHODAS 2.0 5-item “Getting Around” subscale [83], or “Yes” on at least one item of items 1–6 of the Adapted Brief Disability Questionnaire (BDQ) [84]
- Being part of a low-income household as determined by having a yearly household income below the Low Income Limit established by the US Department of Housing and Urban Development’s Fiscal Year Income Limits
- Exhibit a sleep or circadian disturbance as determined by selecting 4 (“quite a bit”) or 5 (“very much”), or the equivalent for reverse-scored items, on one or more of the PROMIS-SD items [85]
- A minimum score of 25 or above on the Montreal Cognitive Assessment (MoCA) as a negative screen for mild cognitive impairment [86]
- Willing and able to give informed consent

#### Exclusion criteria

The exclusion criteria are:

- Severe untreated sleep disordered breathing (apnea-hyponea index; AHI > 30) or moderate untreated sleep disordered breathing (AHI of 15–30) [87] with severe daytime sleepiness (Epworth Sleepiness Scale > 10) [88]. Individuals who screen positive for severe or moderate sleep disordered breathing and provide evidence for adherence to a non-study evidence-based treatment for obstructive sleep apnea (e.g., CPAP) will be eligible to move forward with the study (described in more detail in the screening measures below)
- Medical conditions that prevent a participant from comprehending and following the basic tenants of treatment (e.g., dementia) or that interfere with sleep in a manner that cannot be addressed by a cognitive behavioral treatment (e.g., the Structured Clinical Interview for Sleep Disorders will be used to screen for narcolepsy, REM sleep behavior disorder) or that may preclude full participation (e.g., receipt of end of life care) [89]
- Homelessness
- Night shift work for more than two nights per week in the past 3 months (i.e., regularly scheduled work from 12 a.m. to 6 a.m.)
- Substance abuse/dependence if it makes participation in the study unfeasible. Participants are screened for use, amount and frequency of substance use using questions from the Mini-International Neuropsychiatric Interview (MINI) [90]
- Current suicide risk sufficient to preclude treatment on an outpatient basis (assessed via the Columbia-Suicide Severity Rating Scale) [91]

### Who will take informed consent? {26a}

The assessment team will be responsible for the informed consent process. During the recruitment period, verbal consent will be obtained prior to assessing inclusion and exclusion of potentially eligible patients. Written informed consent will be obtained prior to starting the pre-treatment assessment via HIPAA-compliant DocuSign or paper consent forms. All participants are informed that they can withdraw from the study at any time.

### Additional consent provisions for collection and use of participant data and biological specimens {26b}

The consent form covers all data collected. This trial does not include the collection of biological specimens.

## Interventions

### Explanation for the choice of comparators {6b}

Two variations of TranS-C are tested in the present research: TranS-C + MSI and TranS-C alone. Both treatment groups will complete eight 50-min sessions over 8–10 weeks.

### Intervention description {11a}

#### TranS-C alone

For the present study, TranS-C is comprised of 4 cross-cutting modules featured in every session, 5 core modules, and 8 optional modules that are used based on clinical presentation, treatment goals, and provider case conceptualization [59]. TranS-C modules have been modified and developed to address specific sleep and circadian challenges experienced by midlife and older adults. The *cross-cutting modules* are case formulation, education, motivational enhancement, and goal setting. *Core module 1* targets irregular sleep–wake times, difficulty winding down, and difficulty waking up. *Core module 2* aims to address poor sleep efficiency via stimulus control [92], sleep restriction [93], or sleep compression [94, 95]. *Core module 3* focuses on improving daytime function [96]. *Core module 4* aims to correct unhelpful beliefs about sleep [97]. *Core module 5* promotes the maintenance of change. *Optional module 1* helps participants reduce time in bed for people who are long sleepers. *Optional module 2* addresses delayed or advanced phase circadian problems (e.g., going to sleep later than desired or waking up earlier than desired). *Optional module 3* helps participants manage worries about sleep. *Optional module 4* promotes compliance with Continuous Positive Airway Pressure (CPAP) for participants with sleep apnea [98]. *Optional module 5* helps participants negotiate sleep in complicated environments (e.g., noise from bed/roommates, traffic noise, streetlight entering the bedroom). *Optional module 6* is for participants who experience nightmares [99]. *Optional module 7* is for participants who wish to taper from sleep medications [100]. *Optional module 8* uses problem solving to address sleep challenges for midlife and older adults (e.g., nocturia, chronic pain, medical problems) [101].

### *TranS-C* + *Memory Support*

The TranS-C + MSI condition is identical to the TranS-C alone condition with the exception that Memory Supports will be integrated without adding to the length of the session. Therapists in this condition will be trained to administer an average of 10–12 instances of constructive memory support per session. The rationale is that in our prior research, the optimal dose in which 75% of patients reached a clinically meaningful change ranged from 8 to 12. This dosage of 10–12 is meant as a conservative goal, given that the majority of outcomes were maximized with fewer than 10 memory supports per session [9].

The streamlined MSI used in this study is comprised of 4 constructive memory support strategies: evaluation, categorization, cue-based reminder, and application. Evaluation involves patients evaluating the benefits and drawbacks of a treatment point or comparing the point to an alternative, which promotes deeper processing and conceptual understanding [102, 103]. Categorization is where the participant groups information by higher-order categories, which has been shown to improve recall [104, 105] and increase memory capacity [106, 107]. Cue-based reminders encourage participants to utilize internal and external cues that increase the potential for transfer of knowledge from therapy to everyday life [108]. Application encourages participants to apply learned material in therapy to similar situations [109, 110].

### Criteria for discontinuing or modifying allocated interventions {11b}

Given the minimal risks associated with receiving and discontinuing the intervention, providers are free to discontinue or modify the intervention as they saw fit (i.e., no pre-specified criteria necessary).

### Strategies to improve adherence to interventions {11c}

There are no strategies in the trial to improve adherence, as adherence is not a primary outcome of the study. However, to assess adherence to treatment, the *Therapist Adherence Rating Scale* (TARS) will be used.

### Relevant concomitant care permitted or prohibited during the trial {11d}

No contaminant care measures were taken as the trial was designed to assess the efficacy of the intervention in a real-world community setting.

### Provisions for post-trial care {30}

Given the minimal risk of harm for this study, provisions were not made for ancillary or post-trial care. Details about the Data Safety Monitoring Board can be found elsewhere in the manuscript.

### Outcomes {12}

Table 1 indicates which measures are primary and secondary outcome measures. See Table 2 for the timing of each measure. Participants will complete a consent form and demographics information (age, sex, etc.) in addition to the measures listed below.

**Table 1:** Summary of descriptive, primary and secondary outcomes. Bolded Italics indicate primary outcomes in each domain

Domain	Measures
Descriptive & screening	Demographics; Adapted Brief Disability Questionnaire; Montreal Cognitive Assessment; PROMIS-SD; WHODAS 2.0; Screen for sleep disorders (SCISD, ESS, WatchPAT)
Fidelity	Provider-Rated TranS-C Checklist; Memory Support Treatment Provider Checklist
Adverse Events	Adverse events checklist
**Aim 1**	
Sleep and Circadian Functioning	***PROMIS-SD*** **;** PROMIS-SRI; Sleep Health Composite Score; Sleep Diary (mean sleep efficiency [total sleep time/time in bed X 100]; total wake time (TWT), and total sleep time (TST); Actigraphy (mean TWT, TST, daytime activity)
Daytime Functioning	***Sheehan Disability Scale;*** WHODAS 2.0; ESS
Well-being	***Satisfaction with Life Scale;*** PANAS
**Aim 2**	
Mediators: Patient Memory	Patient Treatment Recall Task (Cumulative recall); Thoughts and Applications Task
**Aim 3**	
Moderators	Demographics (age, education); Montreal Cognitive Assessment; Cognitive Failures Questionnaire; PROMIS-SD/SRI
**Exploratory Aims**	
Adherence	TARS
Credibility	Credibility and Expectancy Questionnaire
Utilization	Utilization Scale
Acceptability, Appropriateness, Feasibility	Acceptability of Intervention Measure; Appropriateness Intervention Measure; Feasibility of Intervention Measure
**Manipulation Checks**	
Memory Support	Memory Support Rating Scale (Total amount; Number of types)
Patient Memory	Patient Treatment Recall Task

*PROMIS-SD* PROMIS-Sleep Disturbance, *WHODAS 2.0* World Health Organization Disability Assessment Schedule, *PANAS SCISD* Structured Clinical Interview for Sleep Disorders, *ESS* Epworth Sleepiness Scale, *PROMIS-SRI* PROMIS-Sleep Related Impairment, *PANAS* Positive Affect and Negative Affect Schedule, *TARS* Therapist Adherence Rating Scale

**Table 2:** SPIRIT depiction of tPliming of study measures

	**Screen**	**Pre-Tx**	**Tx Sessions**				**Post-Tx**	**6FU**	**12FU**
			**1–3**	**4**	**5–7**	**8**			
**Patient**									
Demographic Form	x								
Adapted Brief Disability Questionnaire	x								
Montreal Cognitive Assessment	x								
SCISD^a^	x	x							
WatchPAT	x								
Additional Eligibility Items	x								
PROMIS-SD	x	x					x	x	x
PROMIS-SRI		x					x	x	x
Sleep Health Composite		x					x	x	x
Sleep Diary		x					x	x	x
Actigraphy		x					x		
Sheehan Disability Scale		x					x	x	x
WHODAS 2.0	x	x					x	x	x
Epworth Sleepiness Scale	x	x					x	x	x
Satisfaction with Life Scale		x					x	x	x
PANAS		x					x	x	x
Patient Treatment Recall Task				x			x	x	x
Thoughts and Applications Task				x			x	x	x
Cognitive Failure Questionnaire		x					x	x	x
Credibility and Expectancy			x						
Utilization Scale		x					x	x	x
Adverse Events Checklist							x		
**Therapist**									
Patient Adherence via the TARS			x	x	x	x			
Acceptability, appropriateness, feasibility						x			
Provider-rated TranS-C Checklist			x	x	x	x			
Memory Support Treatment Provider Checklist			x	x	x	x			
**UC Berkeley Team**									
Memory Support Rating Scale	Based on power analysis, one treatment tape per patient will be randomly selected for coding								
TranS-C Fidelity Checklist	Checklist of treatment elements specific to TranS-C will be applied to 10% of tapes								

Additional Eligibility Items include measures for determining inclusion criteria: low-income and exclusion criteria: night shift work, substance use/dependence, current suicide risk, homelessness, and certain medical conditions.

*SCISD* Structured Clinical Interview for Sleep Disorders, *PROMIS-SD* PROMIS-Sleep Disturbance, *PROMIS-SRI* PROMIS-Sleep Related Impairment, *WHODAS 2.0* World Health Organization Disability Assessment Schedule, *PANAS* Positive Affect and Negative Affect Schedule, *TARS* Therapist Adherence Rating Scale, *TranS-C *Transdiagnostic Intervention for Sleep and Circadian Dysfunction.

^a^At screening, the SCISD was used to assess hypersomnolence, narcolepsy, obstructive sleep apnea, and REM sleep behavior disorder. At pre-treatment the SCISD was used to collect diagnostic information pertaining to the presence of the following sleep disorders: insomnia, circadian rhythm sleep–wake disorders, restless leg syndrome, nightmare disorder, and non-REM sleep arousal disorder: sleepwalking and sleep terror types

### Participant measures

Across participant measures, the post-treatment time point is of primary interest.

#### Sleep and circadian functioning

Sleep and circadian functioning will be measured using a multi-method approach employing global, subjective (daily sleep diary), and objective (actigraphy) indices.

The *PROMIS Sleep Disturbance Scale* (PROMIS-SD) is an 8-item scale that will be used to assess perceived sleep problems (e.g., sleep quality, perception of sleep difficulties) over a 7-day period [85]. Items are rated on a 5-point scale ranging from 1 (not at all) to 5 (very much). Sleep disturbance will be measured at each assessment point (pre-treatment, post-treatment, 6FU, and 12FU). T-scores will be calculated from the sum of raw scores using treatment manuals for each timepoint. Higher scores indicate greater sleep disturbance. This scale has demonstrated moderate reliability and validity [85].

The *PROMIS Sleep Impairment Scale* (PROMIS-SRI) is an 8-item scale that will be used to assess perceived sleep problems during waking hours (e.g., alertness, sleepiness, tiredness) over a 7-day period [111]. Sleep impairment will be measured at each assessment point. Items are scored on a 5-point scale ranging from 1 (not at all) to 5 (very much). Higher scores indicate more sleep-related impairment. T-scores will be calculated from the sum of raw scores using treatment manuals for each time point.

The *Composite Sleep Health Score* (CSHS) will be used to calculate a cumulative score that indicates how well someone sleeps. CSHS will be measured at each assessment point. It is comprised of the sum of scores on 6 sleep health dimensions: regularity (midpoint fluctuation), satisfaction (sleep quality question on PROMIS-SD scale), alertness (daytime sleepiness question on PROMIS-SRI scale), timing (mean midpoint), efficiency (sleep efficiency), and duration (total sleep time) [112]. The satisfaction and alertness dimensions are drawn from questions on the PROMIS-SD and PROMIS-SRI scales respectively. All other dimensions are drawn from the daily sleep diary. Each dimension is dichotomized as either 0 = poor or 1 = good. For each dichotomized dimension, we will derive cut-off points for each dimension based on empirical literature and/or recommendations/consensus. Total sum scores range from 0 to 6, where higher scores indicate better sleep health.

The *Consensus Sleep Diary* (CSD) [113] will be used to track subjective sleep for 7 days at each assessment point. The CSD was modified such that the question assessing perceived sleep quality was removed and a question assessing napping time was added (“Did you nap yesterday, if so for how long?”). Daily sleep diaries have been demonstrated to yield a reliable and valid clinical index of sleep and circadian function [114, 115]*.* From the Daily Sleep Diary, mean sleep efficiency (total sleep time/time in bed X 100), total wake time (TWT), and total sleep time (TST) will be calculated.

*Actigraphy* will be used to assess sleep–wake and activity patterns for 7 days at pre-treatment and post-treatment. Participants will wear an ActiGraph wGT3X-BT® (ActiGraph, LLC) which samples movement every 60 s. For actigraphy, mean TWT, TST, and daytime activity will be calculated.

#### Daytime functioning

Three measures will be administered to assess daytime functioning, which are measured at each assessment point. First, the *Sheehan Disability Scale* (SDS) will be used to assess impairment in work/school, social, and family life [116]. Items are rated on a scale of 0 (not at all) to 10 (extremely). Items are summed to produce a single score. Scores range from 0 to 30, with higher scores indicating higher functional impairment. This scale has demonstrated good reliability and validity [116, 117]. Second, the *World Health Organization Disability Assessment. Schedule* (WHODAS 2.0) is a 36-item scale that will be used to assess difficulty in specific areas of functioning during the past 30 days [83]. Items are scored on a 5-point scale from 1 (none) to 5 (extreme or cannot do). Items are summed to produce a single score. Scores range from 36 to 180, with higher scores indicate greater disability. The WHODAS 2.0 has excellent reliability and validity [83, 118]. Third, the *Epworth Sleepiness Scale* (ESS) is an 8-item scale that will be used to measure excessive daytime sleepiness by assessing the likelihood of falling asleep in different situations [88]. Items are scored on a 4-point scale from 0 (would never doze) to 3 (high chance of dozing) and are summed to produce a single score. Scores range from 0 to 24, with scores over 10 reflecting excessive daytime sleepiness.

#### Well-being

Well-being will be assessed with two separate measures, which are assessed at each assessment point. First, the *Satisfaction with Life Scale* (SWLS) is a 5-item instrument that will be used to measure of global satisfaction with one’s life [119]. Items are scored on a 7-point scale ranging from 1 (strongly disagree) to 7 (strongly agree). Items will be summed to produce a single score. Scores range from 5 to 35, with higher scores indicating higher levels of perceived satisfaction with life. The SWLS scale has been found to have excellent reliability and validity [119]. Second, the *Positive Affect and Negative Affect Schedule* (PANAS) will be used to measure emotion and mood [120]. The scale consists of 20 items, with 10 items measuring positive affect (e.g., excited, enthusiastic) and 10 measuring negative affect (e.g., irritable, scared). Items are scored on a 5-point scale ranging from 1 (very slightly or not at all) to 5 (extremely). Items will be summed to produce a single score for positive and negative affect. Scores can range from 10 to 50 for both Positive and Negative Affect, with higher scores representing higher levels of either Positive or Negative affect.

#### Memory assessments

Memory will be assessed using the Patient Treatment Recall Task and the Thoughts and Application Task at mid-treatment (between sessions 4 and 5), post-treatment, 6FU, and 12FU.

The *Patient Treatment Recall Task* will be used to assess participant memory for treatment contents [3]. Participants are required to spend a minimum of 5 min on the recall task and are given up to a maximum of 10 min to recall as many distinct treatment points as possible from all the sessions that they have attended so far (cumulative recall). This task has excellent interrater reliability and, in previous studies, is associated with the amount of memory support received [3, 121]. The overall number of treatment points recalled is determined using a scoring rubric developed in a previous study [3]. The number of treatment points will be summed at each measurement points to produce a single score.

To further assess participant memory and learning, an adapted version of the *Thoughts and Application Task* will be used [122]. The *Thoughts Task* assesses how often participants *thought* about therapy points learned from treatment in the past 24 h. Participants are asked: “In the last 24 h, have the contents covered in your sleep coaching sessions to date come to mind?”, if participants answer no, they are prompted to think for at least 1 min to see if any content comes to mind. If participants answer yes, they are asked “If you were to put a number to it, how many times did the contents covered in your sleep treatment sessions come to mind in the past 24 h?”. Participants are instructed to “write down what contents from treatment came to mind within the last 24 h”. Participants are given a minimum of 1 min and a maximum of 6 min for this task. Similarly, in the *Applications Task* participants are asked if they *applied* any of the treatment points that they learned from the previous sessions in the past 24 h: “In the past 24 h, did you get to apply any of the contents covered in your therapy sessions or use the skills you have been learning during therapy?”, if a participant answers no, they are prompted to think for at least 1 min to see if any content they applied comes to mind. If participants answer yes, they are asked, “If you were to put a number to it, how many times did you apply the contents covered in your sleep treatment sessions in the past 24 h?”. Participants are then instructed to “write down what contents from treatment you were able to apply within the last 24 h”, participants are given a minimum of 1 min and a maximum of 6 min for this task to write down content they have applied in the last 24 h. The overall number of treatment points from the thoughts and applications task will be coded using the same manual as the *Patient Treatment Recall Task.* Number of treatment points from the Thoughts and Applications will be summed separately for each task.

#### Cognitive functioning

The *Cognitive Failures Questionnaire* (CFQ) will be used to assess cognitive functioning at each assessment point. The CFQ is a 25-item scale that specifically assesses failures in perception, memory, and motor function [123]*.* Items are scored on a 5-point scale from 0 (never) to 4 (very often). Items are summed to produce a single score. Scores range from 0 to 100, with higher scores indicating greater cognitive failures.

### Participant exploratory measures

#### Credibility

An adaptation of the *Credibility Expectancy Questionnaire* (CEQ) will be used to assess participants’ perceptions of treatment credibility and outcome expectancy [124]. The CEQ will be measured after treatment session 2. Specifically, the 6-item CEQ was adapted to include only the first four items, assessing the credibility factor fully and the first item of the expectancy factor. In total, four items will be rated via a combination of three 9-point scales (1 = not at all logical/not at all successful/not at all confident, 9 = very logical/very successful/very confident) and one 0–100% scale. Scores from all items will be converted to standardized *z*-scores, and then a total sum CEQ score will be derived, in addition to separate sum scores for the credibility and expectancy factors. Higher scores indicate higher perceived treatment credibility and expected improvement.

#### Utilization

The *Adapted Utilization Scale* will be administered to assess how often sleep health behaviors are performed from TranS-C. The Utilization Scale will be measured at each assessment point. The *Utilization Scale* [125] was modified to address sleep health behaviors relevant to the participants in this study, and to simplify overly complex items, resulting in a 19-item scale. Each sleep behavior is rated on a 5-point scale from 0 (I never use it) to 4 (I always use it). A Total Utilization Treatment Score was created by calculating the mean of all 19 items on the scale at each timepoint separately. Higher scores indicate greater utilization of sleep behaviors learned in treatment.

### Other participant measures

#### Adverse events

The method for assessing adverse events (AEs) follows prior research [126, 127]. A trained assessor will administer the adverse events checklist to clients to assess possible AEs at the post-treatment assessment. *Examples of the questions to be asked are:* I’m going to go through a list of symptoms please let me know whether the sleep changes you made during the sleep coaching treatment that interfered with your normal functioning in anyway? Is the [negative effect] still present? What makes you think that [negative effect] was related to a treatment you received in this study? Then the participant is asked to rate the frequency, intensity and interference caused by the [negative effect] for the past week. Examples of symptoms assessed include low mood, extreme sleepiness, and worsening sleep.

### Additional screening measures

The following measures were used during screening to assess for participant eligibility.

#### Mobility impairment

Experiencing mobility impairment is determined by endorsing “mild”, “moderate”, “severe”, or “extreme or cannot do” on at least one item on the WHODAS 2.0 (described above) 5-item “Getting Around” subscale, or “Yes” on at least one item of items 1–6 of the *Adapted Brief Disability Questionnaire* (BDQ) [84] The BDQ is used to assess whether activities were limited due to health in the prior week (e.g., climbing upstairs or walking uphill). Items are scored on a 3-point scale from 1 (No, not at all) to 3 (Yes, moderately or definitely).

#### Cognitive impairment

The *Montreal Cognitive Assessment* (MoCA) was designed to rapidly detect mild cognitive impairment [86]. Participants complete a variety of tasks that assess attention, memory, executive functioning, language, visuoconstructional skills, and orientation. Scores of these tasks are summed to produce a single score. Scores range between 0 and 30, with scores 26 or above considered normal. More recent research has indicated that a lower cut-off of 25 is optimal for detecting no cognitive impairment [128, 129]. Therefore, a cut-off of 25 was used in the current study to increase the specificity. Participants will be assessed for cognitive impairment remotely using the Full MoCA via Zoom when possible; if participants do not have access to Zoom, the telephone MoCA will be administered [130]. Participants must receive a score of 25 or above to be eligible to participate in the study.

##### Sleep and circadian functioning

The PROMIS-SD has been described above and will also be used as a screening measure to determine if participants exhibit a sleep or circadian disturbance. Participants who select 4 (quite a bit) or 5 (very much) on one or more items will be eligible to participate in the study.

#### Sleep disorders

The *Structured Clinical Interview for Sleep Disorders* (SCISD) is an interview designed to detect the presence of sleep and circadian diagnoses according to the DSM-5 [89]. For screening, the present study included the following diagnoses to be assessed by the SCISD: hypersomnolence disorder, obstructive sleep apnea hypopnea syndrome, REM sleep behavior disorder, and narcolepsy. The presence of REM sleep behavior disorder and narcolepsy is an exclusion criterion for the study as they cannot be addressed by TranS-C. The SCISD will also be used to collect diagnostic information pertaining to the presence of the following sleep disorders at pre-treatment: insomnia, circadian rhythm sleep–wake disorders, restless leg syndrome, nightmare disorder, and non-REM sleep arousal disorder: sleepwalking and sleep terror types.

#### Obstructive sleep apnea

Given the high prevalence of obstructive sleep apnea (OSA) in mid and later life [131, 132] the following steps will be used to address OSA. First, OSA will be screened with the Structured Clinical Interview for Sleep Disorders (SCISD) [89] and the Epworth Sleepiness Scale (ESS) [133]. Individuals who meet the threshold for OSA on the SCISD and score 10 or higher on the ESS will be considered to have “screened positive”. Second, individuals who screen positive and provide evidence for adherence to a non-study evidence-based treatment for OSA (e.g., CPAP) will be eligible to move forward with the study. Third, people who screen positive for OSA with no prior diagnosis or treatment in place will undergo at home sleep apnea testing using WatchPAT® ONE (ZOLL® Itamar®), a disposable home sleep apnea test. Fourth, WatchPAT® ONE results, the sleep apnea module of the SCISD responses, ESS score, and PROMIS-SD responses will be reviewed by MZ, an American Board of Internal Medicine (ABIM) board-certified sleep physician. Participants with a Peripheral Arterial Tonometry-Based Device Apnea Hypopnea Index at the 4% oxygen desaturation level (pAHI 4%) between 15 and 30 will be determined to exhibit moderate OSA and those with a pAHI 4% above 30 will be determined to exhibit severe OSA. Fifth, participants who exhibit severe or moderate untreated OSA will receive a letter addressed to their physician, along with the WatchPAT® ONE results, to assist them in seeking appropriate treatment. Participation will be delayed until treatment has begun and adherence has been established. CPAP adherence is defined as 4 h of use per night on 70% of nights over the past 30 days.

### Therapist exploratory measures

#### Adherence

The *Therapist Adherence Rating Scale* (TARS) is a 5-item scale used by therapists to rate a client’s treatment adherence [134]. This scale will be completed at the end of each treatment session. Each item was rated on a scale from 0 to 100% with 10% increments. A total score will be calculated by averaging all five items, across all eight sessions to produce a single score.

#### Acceptability, appropriateness, and feasibility

Therapists will rate the acceptability, appropriateness, and feasibility of their version of TranS-C using the following 4-item measures: *Acceptability of Intervention Measure* (AIM), *Appropriateness of Intervention Measure* (IAM), and *Feasibility of Intervention Measure* (FIM) [135]. This scale will be completed at the end of each treatment session. All three measures are rated on a 5-point scale from 1 (completely disagree) to 5 (completely agree). A total score will be calculated for AIM, IAM, and FIM by averaging all four items and across all eight sessions.

### Therapist fidelity measures

#### Memory support

The *Memory Support Rating Scale* (MSRS) is a reliable and valid observer-coded measure that assesses the frequency and type of memory support used by treatment providers by analyzing treatment session video recordings [121]. MSRS coders are independent of the treatment provider and assessment teams and are masked to treatment condition. Coders will rate one randomly selected treatment session for each participant. Two measures are derived: the amount of memory support used per session and the number of different types of memory support strategies used per session.

The *Memory Support Treatment Provider Checklist* is a provider-rated measure of the use of the four memory support strategies that comprise the Memory Support Intervention for this study: Application, Categorization, Cue-based Reminder, and Evaluation. Therapists will rate the extent to which they delivered each memory support strategy in a given session on a 4-point scale: never (0 times), a few (1–2 times), often (3–5 times), and many (6 + times). These responses will be scored as never (0 times) = 0, a few (1–2 times) = 1, often (3–5 times) = 2, and many (6 + times) = 3. Two additional variables will be calculated: the total number of times memory support was utilized by summing the scores for each type of memory support on the scale (total number of memory supports used) and the total number of distinct memory support categories used out of a maximum possible total of four (total number of categories of memory support used). As the items on the scale are a range (e.g., 3–4 times), the total number of memory supports used represents a general estimate of memory support used, rather than a precise estimate. Therapists delivering MSI will complete this measure after each treatment session.

#### Treatment content

The *Provider-Rated TranS-C Checklist* is a measure of TranS-C modules delivered during treatment [136]. The original 16-item checklist was adapted to include questions to assess the modules of TranS-C that were included in this study. These additional optional modules include reducing nightmares, tapering hypnotics, and problem solving for sleep challenges. At the end of each treatment session, the therapist will select which items on the list they delivered during that specific session. There is no limit to the number of items a therapist can select for each treatment session. Following previous research in the field [137], a checklist of treatment elements specific to TranS-C was devised. Each element will be rated for presence/absence (i.e., adherence) and quality of delivery (i.e., competence).

#### Treatment fidelity

Each treatment provider will be randomly allocated to deliver only one of the two treatments. The rationale for this decision is that, in prior studies, familiarity with memory supports has made it difficult for therapists to return to treatment-as-usual. Therapists will attend initial training workshops and annual workshops thereafter. In addition, therapists will use a treatment manual [59] and attend weekly supervision meetings. Weekly supervision will be conducted separately for therapists in each condition. Therapists allocated to the memory support condition will attend additional bi-monthly supervision to familiarize themselves with, and practice use of, the memory support strategies.

At the end of each treatment session, all therapists will complete the *Provider-Rated TranS-C checklist* to record the TranS-C modules they delivered [136]. Therapists in the TranS-C + MSI condition will also complete the *Memory Support Treatment Provider Checklist* to record the memory support that they delivered [138]. The *Memory Support Rating Scale* (MSRS) will be used to assess the frequency and type of memory support used by therapists by analyzing one randomly selected session per client [121]. All therapy sessions will be recorded, and a randomly selected subset (10% of sessions) will be closely scrutinized by blind judges using the checklist of treatment elements specific to TranS-C. Fidelity scoring is described in the planned analysis section below.

### Participant timeline {13}

See Table 2 for timing of study measures.

#### Sample size {14}

For Aim 1, sample size was determined by conducting a power analysis using Optimal Design [139, 140] for person-randomized, repeated measures trials with four timepoints (pre-treatment, post-treatment, 6FU, and 12FU). Using a prior dataset comparing CT + MSI and CT-as-usual for depression [25] (R01MH108657), the effect size was calculated for midlife and older adults (≥ 50 years) by averaging the effect size for depression severity at post and 6FU. This approach yielded an effect size of* d* = 0.50. To achieve 80% power, 146 participants are needed. Adding 20% for attrition results in a sample size of 176. For Aim 2, Fritz and MacKinnon [141] provide recommendations for mediation sample size. Using data from R01MH108657 for midlife and older adults (≥ 50 years), the path from the predictor (average constructive memory support) to the mediator (recall at mid-treatment) was small to medium (*f* = 0.18) and the path from the mediator (recall at mid-treatment) to outcome (functional impairment at post-treatment) was medium (*f* = 0.23). Based on these estimates, a sample size of 148 is needed. Adding an additional 20% for attrition results in a sample of 178. The powerMediation package in R [142] was used to calculate the minimum detectable indirect effect of average constructive memory support on outcomes at 6FU and 12FU via recall at mid-treatment. With the proposed sample size of 178 needed for mediation at post-treatment, a minimum standardized indirect effect of 0.56 for outcomes at 6FU and 0.66 for outcomes at 12FU can be detected. The present study focused on powering the primary timepoint of interest, post-treatment. As treatment effects tend to decline over time, we may not be adequately powered to detect statistical significance at 6FU and 12FU. Thus, effect sizes, rather than statistical significance, will be emphasized for the follow-up timepoints. For Aim 3, a minimum detectable effect size difference was calculated via PowerUp! [143]. Estimates from pre-treatment to post-treatment impairment were generated from two prior datasets: (1) midlife and older adults (≥ 50 years), who were randomized to CT + MSI and CT-as-usual [25] (R01MH108657) and (2) midlife and older adults (≥ 50 years), who participated in the pilot trial of TranS-C + MSI [24]. The moderator with the smallest estimates (education from the R01MH108657 trial) was used to help ensure adequate sample size. With an alpha of 0.05, 80% power, and a sample size of 178 (calculated for Aim 2 mediation), the MDES for the moderator effect was *d* = 0.24. Considering that this effect size is comparable to the effect size yielded by similar analyses using prior data (*d* = 0.25 in the R01MH108657 trial), the sample size calculated for Aim 2 will be sufficient to power Aim 3. Together, a minimum sample size of 178 will be sufficient to power all three aims.

### Recruitment {15}

Strategies for achieving adequate participant enrollment include recruiting participants through community-based organizations serving middle-aged and older adults and from the distribution of fliers in the community (e.g., health care clinics, senior centers, libraries).

## Assignment of interventions: allocations

### Sequence generation {16a}

Simple randomization will be used to allocate participants to one of the two treatment arms with a 1:1 allocation ratio. Randomization will be conducted using a computerized, random-number generator in Excel. The resulting random number sequence will be used to assign each subsequent participant to a treatment condition. Randomization is stratified by age (50–69, ≥ 70), as there is evidence that these variables can impact sleep and/or treatment outcomes [60, 144]. The planned stratified randomization is part of the generation of the randomization sequence. We did not stratify by sex as there are no known sex differences in response to either TranS-C or the MSI.

### Concealment mechanism {16b}

Allocation concealment will be ensured as the random number sequence will not be visible to the research team prior to the participant being assigned to a treatment condition.

### Implementations {16c}

The random number sequence will be generated by one project coordinator. Other project coordinators will be responsible for enrolling participants and assigning them to treatment condition.

## Assignment of interventions: blinding

### Who will be blinded {17a}

Therapists delivering TranS-C alone will be blind to the existence of TranS-C + MSI condition. Therapists delivering TranS-C + MSI will be aware of the TranS-C alone condition to prevent them from inadvertently unmasking other team members. Patients will not be aware of their condition assignment. Only the project coordinator involved in the randomization process will know the treatment allocation of each participant. All other team members will be masked to treatment condition, including those conducting assessments, those coding treatment sessions, and biostatisticians.

### Procedure for unblinding if needed {17b}

Unblinding is not expected in the current trial, as both versions of the intervention involve minimal risk and the provider and patient are both privy to the specific treatment modules that have been delivered, emergency unblinding is not applicable.

## Data collection and management

### Plans for assessment and collection of outcomes {18a}

Potential participants will be invited to schedule a full eligibility screening assessment virtually (i.e., via phone or HIPAA-compliant Zoom). After obtaining verbal consent, inclusion and exclusion criteria will be assessed. Participants who screen positive for OSA will be required to follow additional steps to meet eligibility, participants who screen positive for OSA with no prior diagnosis will undergo home sleep apnea testing using WatchPAT® ONE (described in more detail above). Eligible participants will be invited to participate in the pre-treatment assessment. Written informed consent will be obtained prior to starting the pre-treatment assessment via HIPAA-compliant DocuSign or paper consent forms. The pre-treatment assessment, as well as all other assessments, will be conducted virtually over the phone or HIPAA-compliant Zoom. Assessments will be conducted by carefully trained and supervised assessors (i.e., UCB research staff), who enter data into HIPAA-compliant Qualtrics for all assessments. Immediately after the pre-treatment assessment, participants will complete sleep diaries for a 7-day period while wearing an actigraph. Sleep diaries will be completed by participants after receiving a daily scheduled text or email with a link to Qualtrics or collected by an assessor via daily phone calls, who enters the data into Qualtrics.

After participants have completed the pre-treatment assessment including the 7 days of sleep diaries and actigraphy, they will be randomly allocated to TranS-C + MSI or TranS-C alone. Both intervention arms involve eight 50-min individual weekly sessions. Treatment is delivered by therapists (i.e., UCB research staff who are trained and supervised by AGH, a licensed psychologist, and/or EA, a licensed clinical social worker). During treatment, therapists will assist participants in completing one treatment assessment at the end of the second session using Qualtrics to assess the credibility of treatment. Between the fourth and fifth sessions, a mid-treatment assessment consisting of the memory measures will be conducted by assessors virtually. At the conclusion of treatment, typically within 8–10 weeks, the post-treatment assessment will take place within 2 weeks of the final session. Follow-ups will take place 6 months (6FU) and 12 months (12FU) after the start of treatment. The post-treatment assessment, 6FU, and 12FU will be completed by assessors virtually, and anonymized data will be entered into Qualtrics. Actigraphy will be collected for a 7-day period immediately following the post-treatment assessment, and sleep diaries will be collected for a 7-day period after post-treatment assessments, 6FU, and 12FU.

### Plans to promote participant retention and complete follow-up {18b}

Patient retention will be maximized via collaborative efforts between the therapists and assessment team. Considerable efforts will be made by the facilitators and assessors to answer questions and troubleshoot challenges (e.g., scheduling difficulties) to prevent attrition. All participants including those who discontinue treatment will be contacted and asked to complete post-treatment and follow-up assessments.

### Data management {19}

A data management team supervised by the principal investigator, biostatistician (LD), post-docs, and advanced students in clinical psychology on the UC Berkeley team are responsible for downloading, collating, and analyzing the data.

### Confidentiality {27}

All participant-identifiable data will be saved by the assessment team on a secure password-protected and HIPAA-compliant website. Participants and therapists are assigned identification numbers used to link anonymized data that is collected via HIPAA-compliant Qualtrics. When collecting assessments, assessors talk to participants on HIPAA-compliant Zoom or by phone and enter the data into HIPAA-compliant Qualtrics. Participant-identifiable data is not shared with outside entities during or after the trial.

### Plans for collection, laboratory evaluation, and storage of biological specimens for genetic or molecular analysis in this trial/future use {33}

Not applicable to the current trial as no biological specimens will be collected.

## Statistical methods

An alpha = 0.05 will be used for each primary hypothesis. The Benjamini–Hochberg procedure [145] will be used to correct for multiple testing for confirmatory analyses on the primary outcomes (i.e., PROMIS-SD, SDS, SWLS).

### Statistical methods for primary and secondary outcomes {20a}

#### Aim 1: Efficacy of TranS-C + MSI compared to TranS-C alone

Hierarchical Linear Modeling (HLM) [146–148] will be used to test for differences in the trajectories of primary and secondary outcomes across time between the two treatment groups (TranS-C + MSI vs. TranS-C alone). All primary and secondary outcomes are continuous variables (see Table 1) and will be analyzed using the same approach. The 1st level will represent within-person variation and will include dummy-coded time indicators as the predictor (0 = pre-treatment, 1 = post-treatment, 2 = 6FU, 3 = 12FU). The 2nd level will represent between-person variation and will include dummy-coded treatment condition (0 = TranS-C alone, 1 = TranS-C + MSI) and treatment-by-time interaction terms as the predictor. Interactions between treatment condition and the time indicators will be retained only if found significant at the 5% level. A significant interaction between treatment condition and time indicators would suggest that there are different trajectories across time between the two treatment conditions and will be graphed to interpret the interaction. Significant interactions will be interpreted with planned contrasts to assess if treatment effects change over time.

#### Aim 2: Patient memory as a mediator of treatment condition and sleep and circadian functioning

Structural equation modeling (SEM) will be used to test whether patient memory for treatment contents at mid-treatment (i.e., session 4) and post-treatment mediates the relationship between treatment condition (TranS-C + MSI versus TranS-C alone) and the primary patient outcome of sleep disturbance and the secondary patient outcome of sleep-related impairment (measured by the PROMIS-SD and PROMIS-SRI respectively). Three simple mediation models will be specified for each of the two sleep and circadian dysfunction measures at each timepoint (post-treatment, 6FU, 12FU). For each model, the indirect effect of treatment condition to treatment outcome via patient memory will be assessed.

#### Aim 3: Poor treatment response sub-groups as a moderator of treatment condition and sleep and circadian functioning

HLMs will be used to assess the relationship between treatment condition and outcomes at post-treatment, with the following sub-groups included as moderators: age, education, cognitive functioning,^1^ and severity of sleep disruption and sleep-related impairment. These moderators are included in the analysis to assess whether constructive memory support received in the TranS-C + MSI treatment condition results in greater treatment outcomes in any of the sub-groups.

### Interim analyses {21b}

There are no interim analyses planned.

### Methods for additional analyses (e.g., subgroup analyses) {20b}

#### Exploratory Aim 1: Treatment effects on patient adherence, patient-rated treatment credibility, and utilization of treatment elements

An independent samples *t*-test will be used to determine if patient ratings of credibility at the start of treatment differ between TranS-C + MSI compared to TranS-C alone. An independent samples *t*-test will also be used to determine whether average patient adherence during treatment differs between treatment condition. HLMs will be used to determine if participant ratings of their utilization of treatment elements differ by treatment condition from pre- to post-treatment, 6FU, and 12FU.

#### Exploratory Aim 2: Treatment effects on therapist-rated acceptability, appropriateness, and feasibility

Independent samples *t*-tests will be used to determine if therapist ratings of acceptability, appropriateness, and feasibility measured in Session 8 differ between the two treatment conditions (TranS-C + MSI versus TranS-C alone).

#### Exploratory Aim 3: Effects of constructive memory support type on outcomes

HLMs will be used to determine which constructive memory support strategies are associated with the greatest improvement in outcomes at post-treatment, 6FU, and 12FU.

### Fidelity checks

To assess therapist competence and fidelity to treatment, data from the Provider-Rated TranS-C Checklist, checklist of treatment elements specific to TranS-C, and the Memory Support Treatment Provider Checklist will be reported. The Memory Support Treatment Provider Checklist will be used to evaluate whether providers delivered the recommended dose of memory support.

### Manipulation check

To check the assumption that the MSI will effectively result in the delivery of constructive memory support and manipulate patient recall during the TranS-C, independent samples *t*-tests will be used to assess if the total amount of constructive memory support, the different number of types of constructive memory support (measured by the MSRS), and patient recall will be greater in the TranS-C + MSI condition compared to TranS-C alone.

### Methods in analysis to handle protocol non-adherence and any statistical methods to handle missing data {20c}

All analyses will be based on the intent-to-treat principles [149]. Participants who are randomized to treatment condition will be included in the final analyses. We will continue to collect follow-up assessments from participants who withdraw from treatment or are non-adherent to treatment. The *N* by stage of dropout will be reported for the following: dropout after randomization but before the first treatment session, dropout after treatment has begun but before treatment has been completed, and dropout after treatment has been completed but prior to post-treatment, 6- or 12-month follow-up assessments. The number of participants who completed a post-treatment assessment but were lost to 6- and/or 12-month follow-up will also be reported. When available, the reasons for dropout and improvement among participants who dropout will be reported.

For analyses that will use HLM and SEM will use all available data (intent-to-treat). Models will be estimated with maximum likelihood estimation, and missing data will be assumed to be missing at random [149] and has been shown to perform well in simulations of HLM’s with missing data up to 50% [150]. For all other analyses, approaches for handling missing data will be based on the number of missing cases (e.g., listwise deletion vs. multiple imputation).

### Covariates

If dropout is associated with other variables, these variables will be added as predictors to reduce bias. Baseline differences between groups will be examined (e.g., demographics); however, these tests will not be used to select covariates in the primary intent-to-treat analysis. Instead, the potential influences of baseline differences will be evaluated as moderators. The stratification factor (age) will be included as a covariate, per recommendations [151]. Additional covariates may be included if they are associated with the outcome but not the predictor/s. Sex will be included as a covariate in sleep-related outcomes, given the substantial body of literature identifying sex-related differences in sleep problems [80, 152–154]. Presence of OSA and adherence to OSA treatment are likely to influence sleep-related outcomes and will be included as covariates in analyses with these outcomes. Presence of OSA will be dummy-coded as mild and moderate/severe with no diagnosis as the reference group. Adherence to OSA treatment will be dummy-coded as yes with no as the reference group.

### Plans to give access to the full protocol, participant-level data, and statistical code {31c}

Following data collection, de-identified data will be made through the Analysis, Visualization, and Informatics Lab-space (AnVIL).

## Oversight and monitoring

### Composition of the coordinating center and trial steering committee {5d}

This trial is supervised by the principal investigator (AGH), who manages the assessment team, therapists, and the data management team. The principal investigator will meet with members of each team as needed in addition to daily email communication. The responsibilities of each team member are detailed elsewhere in this protocol. In sum, the assessment team will be responsible for the informed consent process and conducting assessments. Therapists are responsible for delivering TranS-C + MSI or TranS-C alone. The data management team will be responsible for downloading, collating, and analyzing the data. There is no coordinating center, trial steering committee, or Stakeholder and Public Involvement Group. The trial sponsor is University of California, Berkeley. Other than ethical approval for the study, the sponsor has no role or ultimate authority in study design; collection, management, analysis, or interpretation of the data; writing of the report; or the decision to submit the report for publication.

### Composition of the data monitoring committee, its role and reporting structure {21a}

A Data Safety Monitoring Board (DSMB) has been formed to monitor participant safety, evaluate the progress of the study, to review procedures for maintaining the confidentiality of data, and the quality of data collection, management, and analyses. The board includes members with expertise in sleep, older adults, basic science, and statistics. Members are independent from the principal investigator and competing interests. A report will be made to the board bi-annually for the first year of the research. In subsequent years, reports will be made to the board annually. However, if safety issues arise, monthly meetings will be conducted. Each report will include a detailed analysis of the study’s progress including the number of participants screened, the number of participants entered, the number of participants dropping out with the reasons for discontinuing, participant descriptive information, and the number of adverse or serious events. Interim analyses will not be conducted.

### Adverse event reporting and harms {22}

In this trial, adverse events (AEs) and serious adverse events (SAEs) are not expected and there is no biological plausibility for this intervention to cause any NIH-defined serious events such as hospitalizations, deaths, or disability. However, these events can occur within the trial population, but they are not anticipated to be related to the trial. Potential harms of treatment may arise from the sleep restriction and stimulus control components of TranS-C, which have been associated with a small amount of short-term sleep deprivation, increased sleepiness, and a deterioration in functioning [155, 156]. Actigraphy can cause minor skin irritation from the watch band, but this is rare. WatchPAT, which screens for sleep apnea, may cause transient psychological distress in some participants, but this is rare. The method for assessing adverse events (AEs) follows prior research [126, 127]. A trained assessor will administer a checklist to patients to systematically assess for possible AEs at the post-treatment assessment. The checklist will screen for unwanted symptoms following treatment and for each endorsed symptom several follow-up questions will be asked (for details, see “Adverse events checklist” under Outcomes). Furthermore, adverse events related to treatment will be assessed by therapists non-systematically throughout treatment. SAEs that are unexpected will be reported to the IRB, NIA program officer, and to the independent DSMB within 48 h of the study’s knowledge of the SAE. All AEs will be reported in future publications, including their nature, severity (mild, moderate, severe), and the relationship to the trial (related, not related).

### Frequency and plans for auditing trial conduct {23}

Organizations not directly involved in the trial (e.g., NIA, Committee for the Protection of Human Subjects, Data Safety Monitoring Board) have the right to audit and, if such a situation arises, will determine the frequency and procedures for auditing.

### Plans for communicating important protocol amendments to relevant parties (e.g., trial participants, ethical committees) {25}

Any protocol changes will be submitted to clinicaltrials.gov and CPHS.

### Dissemination plans {31a}

Results from the trial, as well as analysis code, will be shared via peer-reviewed publications and professional conference presentations. Authorship on future trial publications will be determined according to the guidelines set forth by the American Psychological Association [157].

## Discussion

This study aims to evaluate the integration of the Memory Support Intervention (MSI) into the Transdiagnostic Intervention for Sleep and Circadian Dysfunction (TranS-C) specifically in midlife and older adults. The study protocol will address several research questions. First, we will assess if incorporating the MSI into TranS-C improves sleep and circadian functioning, daytime functioning, and well-being in midlife and older adults. Second, we will test whether the novel treatment target *patient memory* for treatment contents mediates the relationship between treatment condition (TranS-C + MSI versus TranS-C alone) and sleep and circadian outcomes. Third, we will examine if subgroups hypothesized to derive an added benefit from the MSI will moderate the relationship between treatment condition and (a) patient memory for treatment and (b) treatment outcome. Fourth, we will explore whether treatment condition impacts patient adherence, patient-rated credibility/utilization, and therapist-rated acceptability, appropriateness, and feasibility.

This study makes five novel contributions. First, the MSI has the potential to be applied to a broad range of treatment types (pantreatment) and to be utilized for a broad range of mental and physical disorders (transdiagnostic). However, thus far, the MSI has been tested for only one treatment (CT) and one disorder (depression) [25]. The present study is an opportunity to test the broader application of the MSI to treatments beyond CT. Second, the current research protocol assesses the efficacy of a simplified version of the MSI using a more potent and streamlined version of the MSI that emphasizes *constructive* memory support which have been demonstrated to be more effective at improving memory in comparison to other types of memory support [23]. Third, based on past research we have increased the dose of memory support per session to help ensure that a sufficient amount of memory support is delivered to observe clinically meaningful changes in patient outcomes [9]. Fourth, we aim to ensure that memory support is applied following a broader range of treatment contents, as past research has demonstrated that therapists focus on a narrow subset of treatment content [26]. Finally, this is an opportunity to test if the MSI is effective in midlife and older adults who are low-income and have mobility impairments as they may derive a particular benefit from added memory support in treatment as healthy aging is associated with declines in memory function [34–38]. Mid-life and older adults who are low-income and have mobility impairments also face barriers to accessing health care [71, 158], the current study will be delivered over telehealth to improve accessibility and assess the efficacy of the remote delivery of the MSI for this population.

The potential contributions of this study should be considered alongside the protocol’s limitations. First, given that improving sleep can improve learning and memory consolidation [159], it is possible that TranS-C alone will boost patient memory for treatment. However, this is unlikely given that patient memory for treatment did not improve just with TranS-C—in the absence of memory support—in a previous study [160]. Nonetheless, to address this issue, patient memory for treatment will be evaluated in both treatment arms to rule out the possibility that TranS-C alone boosts memory for treatment. Second, patients will complete assessments by either phone or Zoom. To guard against the potential that the different cues available via these communication platforms might impact recall during the memory tasks, patients will be asked to turn off their cameras if using Zoom to better mimic the same conditions as those who take the assessment over the phone. In addition, daytime functioning and well-being were not included as inclusion criteria. Thus, it is possible that participants who have high baseline scores in these domains might mask any potential for improvement in these individuals.

These limitations notwithstanding, testing the efficacy of the MSI integrated into TranS-C has the potential to provide evidence for (a) the efficacy of a new simplified version of the MSI for improving patient memory for treatment with the goal of maintaining health, well-being, and functioning, (b) the wider application of the MSI for midlife and older adults and to the treatment of sleep and circadian problems, (c) the efficacy of treatment interventions delivered via telehealth to improve accessibility, and (d) the efficacy of the MSI for particular sub-groups who are likely to benefit from the intervention.

## Trial status

Protocol version 1, June 2024. Recruitment started in September 2023 and will continue through October 2026.

## Data Availability

Other than the authors and compliance with data-sharing agreements stipulated by the National Institutes of Health, no other entities have contractual agreements to access the final dataset. Deidentified data are submitted to the Analysis, Visualization, and Informatics Lab-space (AnVIL).

## Abbreviations

CBT: Cognitive behavior therapy
MSI: Memory Support Intervention
TranS-C: Transdiagnostic Intervention for Sleep and Circadian Dysfunction
MDD: Major depressive disorder
CT: Cognitive therapy
CBT-I: Cognitive Behavior Therapy for Insomnia
CPAP: Continuous Positive Airway Pressure
CPHS: Committee for the Protection of Human Subjects at the University of California, Berkeley
PROMIS-SRI: PROMIS Sleep-Related Impairment Scale
PROMIS-SD: PROMIS Sleep-Disturbance Scale
CSD: Consensus Sleep Diary
CSHS: Composite Sleep Health Score
SCISD: Structured Clinical Interview for Sleep Disorders
SDS: Sheehan Disability Scale
WHODAS: World Health Organization Disability Assessment Schedule
BDQ: Adapted Brief Disability Questionnaire
ESS: Epworth Sleepiness Scale
SWLS: The Satisfaction with Life Scale
PANAS: Positive Affect and Negative Affect Schedule
CFQ: Cognitive Failures Questionnaire
MoCA: The Montreal Cognitive Assessment
OSA: Obstructive sleep apnea
AHI: Apnea Hypopnea Index
AE: Adverse events
CEQ: Credibility Expectancy Questionnaire
MSRS: Memory Support Rating Scale
TARS: Therapist Adherence Rating Scale
AIM: Acceptability of Intervention Measure
IAM: Appropriateness of Intervention Measure
FIM: Feasibility of Intervention Measure
UCB: University of California, Berkeley
MS: Memory Support
DSMB: Data Safety Monitoring Board
AnVIL: Analysis, Visualization, and Informatics Lab-space

## Footnotes

1 The Montreal Cognitive Assessment (MoCA) was not used to as a moderator addressing cognitive functioning as participants were excluded from the study based on this measure which would have resulted in reduced variability across participants. In contrast, the Cognitive Failures Questionnaire (CFQ) will be used as a moderator variable assessing cognitive functioning as the CFQ captures a wider range of cognitive challenges that midlife and older adults without MCI may experience.
